# Sleep deprivation and immunoglobulin E level

**DOI:** 10.3389/fmed.2022.955085

**Published:** 2022-11-16

**Authors:** Shih-Wei Yang, Hui-Fang Yang, Yuan-Yuei Chen, Wei-Liang Chen

**Affiliations:** ^1^Division of Plastic and Reconstruction Surgery, Department of Surgery, Tri-Service General Hospital, National Defense Medical Center, Taipei, Taiwan; ^2^Division of Family Medicine, Department of Family and Community Medicine, Tri-Service General Hospital, School of Medicine, National Defense Medical Center, Taipei, Taiwan; ^3^Division of Geriatric Medicine, Department of Family and Community Medicine, Tri-Service General Hospital, School of Medicine, National Defense Medical Center, Taipei, Taiwan; ^4^Division of Family Medicine, Department of Community Medicine, Taoyuan Armed Forces General Hospital, Taoyuan, Taiwan

**Keywords:** sleep, sleep disorders, sleep deprivation, serum immunoglobulin E level, sleep duration

## Abstract

**Objectives:**

Sleep deprivation is a common issue for modern people and leads to many morbidities and mortality. Some papers also suspected the relationship between Immunoglobulin E (IgE) level and sleep deprivation. The purpose of this article is to make the vicious circle between serum IgE level and sleep deprivation clear.

**Materials and methods:**

In this study, we included 4,460 population aged around 48 years old respective 2,145 males and 2,315 females enrolled in the National Health and Nutrition Examination Survey (NHANES). Sleep durations were recorded, in hours, in whole numbers by the participants. The levels of total IgE were measured by anti-IgE. All procedures were analyzed using SPSS version 18 (SPSS, Inc., Chicago, IL, USA).

**Result:**

The statistical significance between higher IgE level and **≤**5 h sleep duration was noted (Beta coefficients: 64.04, 95% confidence interval (CI): 2.35, 125.72, *P* = 0.042). In sex difference, the correlation between short sleep duration and high serum IgE levels was noted in male [Beta coefficients: 120.225 (*P* = 0.008)] but not in female. There were no positive findings in the ethnicity-based correlation between serum IgE levels and sleep duration.

**Conclusion:**

This study indicated that short sleep duration (**≤**5 h) is associated with higher serum IgE levels, especially in men. Further longitudinal investigations concerning the effect of sleep deprivation on serum IgE might provide a better explanation for the pathophysiology underlying autoimmune disease and sleep deprivation.

## Introduction

Sleep has an important role to play in the human immune system and is critical for the restoration and maintenance of homeostasis (1). Sleep deprivation is a common issue in modern life, leading to the impaired quality of life for patients and their families (2). Several different scales are used to measure sleep quality. The Epworth sleepiness scale is a short easy way to measure sleepiness in ordinary life situations. Users rate their chances of sleeping in eight situations on a 4-point scale (3). The Pittsburgh sleep quality index (PSQI) is composed of 19 items classified into seven components: subjective sleep quality, sleep latency, sleep duration, habitual sleep efficiency, sleep disturbances, use of sleeping medication, and daytime dysfunction during the past month (4). To date, many different reasons of sleep deprivation have been reported in the literature. Stimuli such as stress or anxiety may be the cause of sleep deprivation (5). Hormone, such as melatonin, is involved in the regulation of the human sleep–wake cycle and circadian rhythm (6). Melatonin and melatonin receptor agonists have been shown to be therapeutic agents for the treatment of circadian rhythm sleep disorders and some type of insomnia (7). Metabolic disease has also been linked to sleep deprivation in several studies (8). An inadequately short sleep duration may lead to mortality and morbidity (9). A previous study confirmed that rheumatoid arthritis (RA) patients have severe sleep fragmentation compared to the control group (10). Seventy-five percent of primary Sjögren’s syndrome patients had moderate or severe sleep disturbances in a cross-sectional study (11). In 1995, one study showed that the systemic lupus erythematosus (SLE) group had greater overall fatigue and longer sleep latency and total sleep time than the control groups (12).

Immunoglobulin E (IgE) is a type of antibody, which is monomeric and consists of four constant regions, in contrast to other immunoglobulins that contain only three constant regions (13). Typically, IgE is the least abundant Ig isotype, with a concentration of ∼150 ng/mL in the sera of healthy individuals (14). There are two types of IgE molecules: free IgE produced by plasma cells and membrane-bound IgE maintained on the surface of B cells (15). IgE plays an essential role in the immunity against parasites and in type 1 hypersensitivity, which manifests in allergic diseases, including allergic asthma, allergic rhinitis, food allergies, and some types of atopic dermatitis (16, 17). More recently, IgE autoantibodies have been found to participate in the damaging immune responses that characterize autoimmunity (18). It is now recognized that SLE, RA, bullous pemphigoid (BP), and chronic urticaria are most likely mediated by IgE autoantibodies (19).

To date, it is unclear whether a vicious circle links sleep deprivation and serum IgE level. Our purpose is to identify the association between levels of serum IgE and sleep deprivation by analyzing information from the National Health and Nutrition Examination Survey (NHANES).

## Materials and methods

### Study design and participants

The NHANES data consisted of a comprehensive interview and a health examination conducted by the National Center for Health Statistics (NCHS). We conducted a cross-sectional study using the NHANES database in 2001–2004 period. The exclusion criteria included individuals without complete information about laboratory results or clinical examinations. In this study, we included 4,460 individuals–2,145 males and 2,315 females, approximately 48 years of age.

### Measurement of sleep duration

Sleep duration was evaluated with the measure item, “On average, for how long do you usually sleep at night on weekdays or workdays?” Answers were categorized as whole numbers into five groups (≤5, 6, 7, 8, and ≥9 h) for analysis by the participants.

### Assessment of covariates

We collected participant information on age, sex and race-ethnicity (including Mexican American, non-Hispanic white, and non-Hispanic black). Smoking status was categorized as “non-smoker” for subjects who never smoked and “smoker” for subjects who were ex-smokers or current smokers by asking the question, “Do you now smoke cigarettes?” Alcohol drinking was determined through the participants’ self-reports. Co-morbidities, including asthma, congestive heart failure, coronary heart disease, angina, and stroke, were ascertained by self-reports. The total IgE level was measured using anti-IgE, which was covalently coupled to the ImmunoCap™ reaction vessel. CRP levels were measured by latex-enhanced nephelometry using the Behring Nephelometer System. Total bilirubin levels were evaluated by biochemical profiling. The levels of total cholesterol, triglycerides, and HDL-C were assessed using the Hitachi-704 analyzer. Creatine was assessed using the Beckman UniCel^®^ DxC800 Synchron. Fasting glucose was assessed using the Hexokinase-mediated reaction Roche/Hitachi Cobas C Chemistry Analyzer. Insulin was assessed with human insulin immunoassay by using the Molecular Devices, SpectraMax 250. Vitamin B12 are measured by using the Bio-Rad Laboratories.

### Statistical analysis

All procedures were analyzed using SPSS version 18 (SPSS, Inc., Chicago, IL, USA). Descriptive information related to continuous and categorical covariates was presented as mean ± standard deviation and number (%). The comparison of characteristics and covariates across subgroups was performed using ANOVA for continuous variables and the chi-squared test for categoric variables. We analyzed the association between sleep duration and serum IgE levels using a multivariate linear regression model. Three models were executed for covariate calibration. Model 1 was adjusted for age, gender, and race. Model 2 was model 1 along with adjustment for body mass index (BMI), CRP, total bilirubin, total cholesterol, triglycerides, HDL, creatine, fasting glucose, insulin, vitamin B12. Model 3 was Model 2 along with adjustment for sleep disorders, snorting/stop breathing, mental health service use, asthma, congestive heart failure, coronary heart disease, angina, stroke, cancer, smoking, and alcohol drinking.

## Results

### Sample characteristics

A total of 4,480 participants, for whom serum IgE level data were available and who reported their sleep duration from 2001 to 2004, were enrolled from the NHANES database. Table 1 presents the clinical characteristics and demographic data of the participants classified by sleep duration.

**TABLE 1:** Characteristics of study participants.

	Sleep duration at night						
	
Characteristic	≤5 h *N* = 673	6 h *N* = 1,001	7 h *N* = 1,190	8 h *N* = 1,232	≥9 h *N* = 364	Total *N* = 4460	*P-value*
Continuous variables*							
Age (years)	47.84 (17.87)	48.16 (17.92)	46.97 (17.75)	49.49 (19.98)	50.72 (22.42)	48.36 (18.88)	0.002
BMI (kg m^–2^)	30.22 (7.60)	29.17 (6.77)	28.23 (5.79)	28.35 (6.41)	28.59 (8.67)	28.80 (6.77)	<0.001
Serum total IgE Ab (kU/L)	196.63 (440.10)	182.30 (676.33)	151.36 (396.09)	144.03 (403.55)	160.46 (377.84)	163.85 (480.19)	0.11
CRP (mg/dL)	0.51 (0.70)	0.48 (0.84)	0.42 (0.86)	0.51 (0.88)	0.59 (0.89)	0.49 (0.84)	0.008
Total bilirubin (mg/dL)	0.70 (0.28)	0.70 (0.29)	0.73 (0.48)	0.69 (0.28)	0.67 (0.28)	0.70 (0.35)	0.042
Total cholesterol (mg/dL)	198.39 (46.06)	199.04 (40.74)	201.70 (45.22)	202.58 (43.87)	200.66 (44.69)	200.77 (43.98)	0.193
Triglyceride (mg/dL)	154.86 (130.60)	145.78 (101.16)	157.79 (141.20)	160.06 (120.54)	155.14 (94.24)	155.07 (122.22)	0.079
HDL cholesterol (mg/dL)	53.96 (16.91)	54.69 (15.92)	55.36 (17.04)	55.63 (17.18)	56.29 (16.81)	55.15 (16.80)	0.139
Creatinine (mg/dL)	0.95 (0.45)	0.97 (0.42)	0.92 (0.30)	0.94 (0.58)	0.92 (0.33)	0.94 (0.44)	0.133
Fasting Glucose (mg/dL)	106.20 (34.92)	105.16 (35.18)	101.67 (22.52)	106.06 (36.74)	107.44 (40.93)	104.89 (33.46)	0.116
Insulin (μU/mL)	12.86 (11.98)	12.47 (11.35)	12.47 (18.46)	11.87 (12.27)	10.91 (9.35)	12.24 (13.69)	0.529
Vitamin B12 (pg/mL)	637.40 (2742.34)	602.50 (1494.84)	598.25 (1465.21)	577.04 (806.48)	530.57 (407.01)	593.79 (1549.13)	0.864
**Categorical variables^†^**							
Male	344 (51.1)	495 (49.5)	581 (48.8)	583 (47.3)	142 (39.0)	2145 (48.1)	0.003
Race-ethnicity							<0.001
Mexican American	124 (18.4)	196 (19.6)	238 (20.0)	280 (22.7)	69 (19.0)	907 (20.3)	
Other Hispanic	21 (3.1)	31 (3.1)	46 (3.9)	33 (2.7)	6 (1.6)	137 (3.1)	
Non-Hispanic White	256 (38.0)	446 (44.6)	666 (56.0)	678 (55.0)	202 (55.5)	2248 (50.4)	
Non-Hispanic Black	245 (36.4)	284 (28.4)	193 (16.2)	202 (16.4)	71 (19.5)	995 (22.3)	
Sleep disorders	100 (14.9)	67 (6.7)	59 (5.0)	59 (4.8)	21 (5.8)	306 (6.9)	<0.001
Snorting/Stop breathing	160 (26.7)	223 (24.4)	189 (17.1)	192 (17.2)	56 (17.0)	820 (20.2)	<0.001
Mental health service use	68 (10.1)	59 (5.9)	75 (6.3)	81 (6.6)	38 (10.4)	321 (7.2)	0.001
Asthma	121 (18.0)	135 (13.5)	132 (11.1)	142 (11.5)	44 (12.1)	574 (12.9)	<0.001
Congestive heart failure	38 (5.6)	31 (3.1)	20 (1.7)	44 (3.6)	15 (4.1)	148 (3.3)	<0.001
Coronary heart disease	33 (4.9)	43 (4.3)	28 (2.4)	58 (4.7)	13 (3.6)	175 (3.9)	0.052
Angina/Angina pectoris	32 (4.8)	31 (3.1)	29 (2.4)	34 (2.8)	11 (3.0)	137 (3.1)	0.116
Stroke	36 (5.3)	41 (4.1)	34 (2.9)	34 (2.8)	27 (7.4)	172 (3.9)	<0.001
Cancer/Malignancy	47 (7.0)	68 (6.8)	99 (8.3)	110 (8.9)	46 (12.6)	370 (8.3)	0.001
Smoking	339 (50.4)	486 (48.6)	542 (45.5)	575 (46.7)	171 (47.0)	2113 (47.4)	0.453
Alcohol drinking	394 (62.9)	646 (70.1)	801 (71.7)	789 (69.1)	220 (64.9)	2850 (68.7)	0.003

*Continuous variables are presented as mean (standard deviation).

^†^Categorical variables are presented as number (percentage).

BMI, body mass index; IgE, Immunoglobulin E; Ab, Antibody; CRP, C-reactive protein; HDL, high-density lipoprotein.

The mean age was 48.36 ± 18.88 years, and 48% of the participants were male. The race/ethnicity also presented a distinctive distribution.

### Association between immunoglobulin E level and sleep duration

The relationship between the IgE level and sleep deprivation is shown in Table 2. We used 7 h sleep duration as the reference group. After we adjusted covariates in model 2 and model 3, the β coefficients of ≤5 h of sleep duration were 64.84 [95% confidence interval (CI): 3.93, 125.75, *P* = 0.037] and 64.04 (95% CI: 2.35, 125.72, *P* = 0.042). Compared to other groups, higher levels of serum IgE were significantly associated with a sleep duration ≤5 h; the *P* < 0.05 for sleep duration ≤5 h after adjusting the covariates. However, the *P*-values were >0.05 in individuals whose sleep duration was 6, 8, and ≥9 h.

**TABLE 2:** Association between different sleep duration and immunoglobulin E (IgE) level.

Models*	Sleep duration^†^	β^‡^ (95% CI)	*P*
Model 1	≦5 h 6 h 7 h 8 h ≧9 h	71.74 (11.20, 132.29) 41.13 (−14.16, 96.41) reference −6.02 (−57.80, 45.77) 3.78 (−70.97, 78.54)	0.020 0.145 − 0.820 0.921
Model 2	≦5 h 6 h 7 h 8 h ≧9 h	64.84 (3.93, 125.75) 37.48 (−17.98, 92.94) reference −6.23 (−58.35, 45.88) 2.91 (−72.18, 77.99)	0.037 0.185 − 0.815 0.939
Model 3	≦5 h 6 h 7 h 8 h ≧9 h	64.04 (2.35, 125.72) 36.91 (−18.77, 92.59) reference −8.67 (−60.93, 43.58) 4.34 (−71.05, 79.73)	0.042 0.194 − 0.745 0.910

*Adjusted covariates:

Model 1 = age, gender, race/ethnicity.

Model 2 = Model 1 + (BMI, CRP, total bilirubin, total cholesterol, triglycerides, HDL, creatine, fasting glucose, insulin, vitamin B12).

Model 3 = Model 2 + (sleep disorders, snorting/stop breathing, mental health service use, asthma, congestive heart failure, coronary heart disease, angina, stroke, cancer) + (smoking, alcohol drinking).

^†^Subjects with 7-h sleep length were the reference group.

^‡^β coefficient can be interpreted as differences in the mean homocysteine comparing subjects in the other 4 groups of sleep duration to those in the 7-h sleep length.

IgE, Immunoglobulin E; BMI, Body Mass index; CRP, C-reactive protein; HDL, high-density lipoprotein.

### Correlation of sex and ethnicity with sleep duration and serum immunoglobulin E levels

**FIGURE 1:**
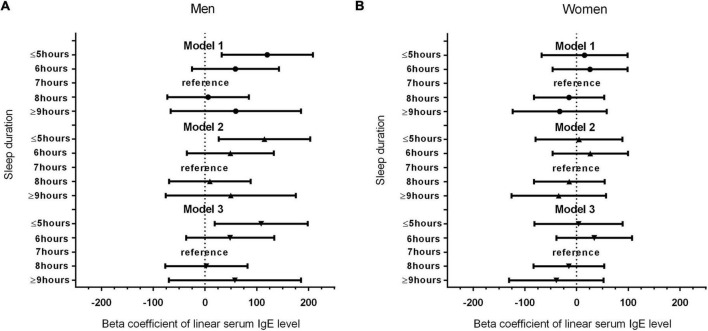
**(A)** Association between sleep duration and serum IgE level in male participants. **(B)** Association between sleep duration and serum IgE level in female participants.

Figure 1 shows the results of our analysis of the correlation of sex with serum IgE levels and sleep duration. Interestingly, compared with the reference group (7-h sleep duration), the shortest sleep duration (≤5 h) in model 1 had regression coefficients of 120.225 (*P* = 0.008) for men and 15.332 (*P* = 0.718) for women. In different models, the trend with the shortest sleep duration and elevated serum IgE levels remained unchanged in men (*P* < 0.05) but not in women, even after adjusting all the covariates. Table 3 shows the ethnicity-based correlation between sleep duration and serum IgE levels. There were no positive findings in the Mexican American, Other Hispanic, non-Hispanic White, and non-Hispanic black groups even after we adjusted for several covariates in models 2 and 3.

**TABLE 3:** Race specific association between sleep duration and serum immunoglobulin E (IgE) level.

		Mexican American		Other Hispanic		Non-Hispanic white	
						
Models*	Sleep duration^†^	β^‡^ (95% CI)	*P*	β^‡^ (95% CI)	*P*	β^‡^ (95% CI)	*P*
Model 1	≦5 h 6 h 7 h 8 h ≧9 h	−17.439 (−155.482, 120.604) −1.173 (−123.575, 121.229) reference 11.495 (−99.108, 122.097) −93.054 (−258.782, 72.675)	0.804 0.985 − 0.838 0.270	−130.681 (−342.533, 81.172) −139.397 (−331.660, 52.866) reference −99.996 (−312.198, 112.205) −280.693 (−64.547, 625.932)	0.221 0.151 − 0.348 0.109	53.993 (−11.237, 119.223) 13.254 (−44.178, 70.685) reference −4.199 (−54.091, 45.694) 48.928 (−23.118, 120.974)	0.105 0.651 − 0.869 0.183
Model 2	≦5 h 6 h 7 h 8 h ≧9 h	−1.385 (−138.373, 135.602) −5.310 (−129.620, 118.999) reference −6.153 (−119.944, 107.638) −86.109 (−252.198, 79.981)	0.984 0.933 − 0.915 0.309	−112.991 (−335.184, 109.202) −182.513 (−392.017, 26.991) reference −104.089 (−362.034, 153.855) 342.078 (−26.371, 710.526)	0.310 0.086 − 0.419 0.068	49.467 (−16.332, 115.265) 11.077 (−46.605, 68.758) reference −7.777 (−57.954, 42.400) 41.956 (−30.656, 114.569)	0.140 0.706 − 0.761 0.257
Model 3	≦5 h 6 h 7 h 8 h ≧9 h	6.160 (−136.460, 148.781) −18.058 (−146.095, 109.979) reference −1.054 (−117.759, 115.652) −84.688 (−253.309, 83.932)	0.932 0.782 − 0.986 0.324	−273.604 (−548.544, 1.336) −281.914 (−528.446, −35.381) reference −138.006 (−422.746, 146,733) 425.882 (−9.677, 861.440)	0.051 0.026 − 0.330 0.055	34.725 (−31.574, 101.023) 4.147 (−53.459, 61.754) reference −8.687 (−58.521, 41.146) 44.137 (−28.562, 116.836)	0.304 0.888 − 0.732 0.234

		**Non-Hispanic black**		**Others**	
				
Models*	**Sleep duration** ^†^	**β^‡^ (95% CI)**	***P***	**β^‡^ (95% CI)**	***P***

Model 1	≤5 h 6 h 7 h 8 h ≥9 h	88.241 (−89.656, 266.139) 108.650 (−68.053, 285.352) reference −36.265 (−226.652, 154.122) −47.163 (−323.467, 229.141)	0.330 0.227 − 0.708 0.737	520.806 (63.728, 977.884) 13.358 (−311.690, 338.407) reference −8.973 (−355.247, 337.301) −78.890 (−485.328, 327.549)	0.026 0.935 − 0.959 0.700
Model 2	≤5 h 6 h 7 h 8 h ≥9 h	70.723 (−111.160, 252.606) 106.895 (−72.673, 286.463) reference −39.714 (−233.874, 154.446) −51.406 (−331.712, 228.899)	0.445 0.242 − 0.688 0.719	621.197 (121.578, 1120.816) 17.856 (−337.593, 373.306) reference 65.887 (−310.837, 442.611) −97.807 (−537.231, 341.616)	0.016 0.920 − 0.728 0.658
Model 3	≤5 h 6 h 7 h 8 h ≥9 h	55.327 (−131.194, 241.847) 100.367 (−79.665, 280.399) reference −68.470 (−263.526, 126.586) −80.437 (−363.387, 202.513)	0.560 0.274 − 0.490 0.576	713.323 (90.219, 1336.426) 68.785 (−357.429, 494.999) reference 60.213 (−360.114, 480.539) −148.380 (−656.653, 359.894)	0.026 0.747 − 0.775 0.560

*Adjusted covariates:

Model 1 = age, race/Ethnicity.

Model 2 = Model 1 + (BMI, CRP, total bilirubin, total cholesterol, triglycerides, HDL, creatine, fasting glucose, insulin, vitamin B6 and B12, folate).

Model 3 = Model 2 + (sleep disorders, snorting/stop breathing, mental health service use, asthma, congestive heart failure, coronary heart disease, angina, stroke, cancer) + (smoking, alcohol drinking).

^†^Subjects with 7-h sleep length were the reference group.

^‡^β coefficient can be interpreted as differences in the mean homocysteine comparing subjects in the other 4 groups of sleep duration to those in the 7-h sleep length.

IgE, Immunoglobulin E; BMI, Body Mass index; CRP, C-reactive protein; HDL, high-density lipoprotein.

## Discussion

After analyzing the data, which represented a sample of the US adult population, we found that short sleep duration (≤5 h) was associated with high IgE levels. The association remained significant even after we adjusted different covariates (*P* < 0.05). Conversely, there was no association between longer sleep duration and IgE levels. Further, the association between the shortest sleep duration (≤5 h) and elevated serum IgE levels was observed in men but not women. Our study is the first observational study with a large sample size, stratified by sex and race/ethnicity, to analyze the relationship between sleep duration and serum IgE levels.

Direct evidence of the relationship between sleep duration and serum IgE levels was limited. One review article published in 2015 indicated that sleep deprivation may induce the onset of autoimmune disease (20). A large cohort study by Hsiao et al. indicated that the overall risk for incident autoimmune diseases was significantly higher in patients with non-apnea sleep disorders (NSD) [hazard ratio (HR) = 1.47, 95% CI = 1.41–1.53] (21). Another cohort study found that the hazard ratio (95% CI = 1.32–2.77, *p* < 0.001) for the development of autoimmune disease during a five-year follow-up period was 1.91 times greater for patients with obstructive sleep apnea (OSA) than others (22).

Our study had some noteworthy findings. First, we found out that only men showed a significant association between high serum IgE levels and short sleep duration (≤5 h). Sex hormones are known to affect the immune system and contribute to sex-based differences in immune-mediated disease responses. Testosterone inhibits immune responses, whereas estrogen and progesterone tend to enhance immune responses (23). The immune-enhancing effects of sex hormones tend to increase the predisposition of women to several autoimmune diseases, such as multiple sclerosis, SLE, and RA (24). Further, several studies suggested that, both as children and adults, men have higher IgE levels than women [odds ratio (OR) 95% CI: 1.6 (1.4–1.8, *p*-value < 0.001)] (25). Thus, the levels of IgE and the prevalence of sensitization was higher in men than women (26). As a result, although the prevalence of auto-immune disease was higher in women, the serum IgE levels in men played a more important role than in women, contributing to the results of our study.

Second, our analysis revealed that there was no difference in the association between serum IgE levels and sleep duration in different racial groups. The evidence for an association between sleep duration and serum IgE levels by race/ethnicity is limited. One study reported that serum IgE levels vary by age, occupation, environment, and race/ethnicity (27). As the association between ethnicity and sleep duration is reported to be unclear, more studies were needed to better understand the role of ethnicity in sleep duration and serum IgE levels (28).

Our study has a few limitations. First, this was a cross-sectional observational study. Further cohort studies are needed to confirm our findings. Second, because sleep duration was evaluated by self-reported questionnaires, recall bias cannot be ignored. Third, there were several important unmeasured confounding factors that may influence these results. For example, recent psychosocial stress and individual lifestyles might influence sleep duration but were not considered in our analyses. Finally, the baseline serum IgE level might vary by ethnicity, nutritional support, environment, and genetics. Thus, the results of these correlations between serum IgE levels and various factors must be interpreted carefully.

## Conclusion

Our study indicated that sleep deprivation (sleep duration ≤5 h) is associated with higher serum IgE levels, especially in men. High serum IgE levels may increase the risk of autoimmune disease. Further longitudinal investigations concerning the effect of sleep deprivation on serum IgE might provide a better explanation for the pathophysiology underlying autoimmune disease and sleep deprivation.

## Data availability statement

The original contributions presented in this study are included in the article/supplementary material, further inquiries can be directed to the corresponding author.

## Ethics statement

The studies involving human participants were reviewed and approved by Protocol #98-12. The patients/participants provided their written informed consent to participate in this study.

## Author contributions

S-WY and W-LC designed the initial study and responsible for the decisions of data analysis. S-WY also managed and retrieved the data, contributed to primary data analysis and explanation, and drafted the initial script. S-WY, H-FY, Y-YC, and W-LC decided on the methods of data collection. W-LC conceptualized the study, inspected all sides of the study, critically reviewed and revised the initial script, and approved the final manuscript as submitted. All authors meet the ICMJE criteria for authorship.

## Abbreviations

IgE: immunoglobulin E
RA: rheumatoid arthritis
SLE: systemic lupus erythematosus
BP: bullous pemphigoid
NHANES: National Health and Nutrition Examination Survey
NCHS: National Center for Health Statistics
CI: confidence interval
NSD: non-apnea sleep disorders
HR: hazard ratio
OSA: obstructive sleep apnea
OR: odds ratio
IRB: Institutional Review Board.

## References

[1] Besedovsky L Lange T Haack M. The sleep-immune crosstalk in health and disease. *Physiol Rev.* (2019) 99:1325–80. 10.1152/physrev.00010.2018 30920354 PMC6689741

[2] Karacan I Thornby JI Anch M Holzer CE Warheit GJ Schwab JJ Prevalence of sleep disturbance in a primarily urban Florida County. *Soc Sci Med.* (1976) 10:239–44. 10.1016/0037-7856(76)90006-8 968513

[3] Doneh B. Epworth sleepiness scale. *Occup Med.* (2015) 65:508. 10.1093/occmed/kqv042 26240130

[4] Buysse DJ Reynolds CF III Monk TH Berman SR Kupfer DJ. The Pittsburgh Sleep Quality Index: a new instrument for psychiatric practice and research. *Psychiatry Res.* (1989) 28:193–213. 10.1016/0165-1781(89)90047-4 2748771

[5] Schutte-Rodin S Broch L Buysse D Dorsey C Sateia M. Clinical guideline for the evaluation and management of chronic insomnia in adults. *J Clin Sleep Med.* (2008) 4:487–504. 10.5664/jcsm.27286 18853708 PMC2576317

[6] Uchiyama M. Melatonin receptor agonist. *Nihon Rinsho.* (2015) 73:1017–22. 26065135

[7] Mishima K. Melatonin as a regulator of human sleep and circadian systems. *Nihon Rinsho.* (2012) 70:1139–44. 22844795

[8] Nedeltcheva AV Scheer FA. Metabolic effects of sleep disruption, links to obesity and diabetes. *Curr Opin Endocrinol Diabetes Obes.* (2014) 21:293–8. 10.1097/MED.0000000000000082 24937041 PMC4370346

[9] Gangwisch JE Heymsfield SB Boden-Albala B Buijs RM Kreier F Opler MG Sleep duration associated with mortality in elderly, but not middle-aged, adults in a large US sample. *Sleep.* (2008) 31:1087–96. 18714780 PMC2542954

[10] Hirsch M Carlander B Vergé M Tafti M Anaya JM Billiard M Objective and subjective sleep disturbances in patients with rheumatoid arthritis. A reappraisal. *Arthritis Rheum.* (1994) 37:41–9. 10.1002/art.1780370107 8129763

[11] Tishler M Barak Y Paran D Yaron M. Sleep disturbances, fibromyalgia and primary Sjögren’s syndrome. *Clin Exp Rheumatol.* (1997) 15:71–4. 9093776

[12] McKinley PS Ouellette SC Winkel GH. The contributions of disease activity, sleep patterns, and depression to fatigue in systemic lupus erythematosus. A proposed model. *Arthritis Rheum.* (1995) 38:826–34. 10.1002/art.1780380617 7779127

[13] Wu LC Zarrin AA. The production and regulation of IgE by the immune system. *Nat Rev Immunol.* (2014) 14:247–59. 10.1038/nri3632 24625841

[14] King CL Poindexter RW Ragunathan J Fleisher TA Ottesen EA Nutman TB. Frequency analysis of IgE-secreting B lymphocytes in persons with normal or elevated serum IgE levels. *J Immunol.* (1991) 146:1478–83. 1899687

[15] McCoy KD Harris NL Diener P Hatak S Odermatt B Hangartner L Natural IgE production in the absence of MHC Class II cognate help. *Immunity.* (2006) 24:329–39. 10.1016/j.immuni.2006.01.013 16546101

[16] Hofman T Hanasz-Jarzynska T. Based on normal levels of total IgE can diagnosis of atopic diseases in children be ruled out?. *Pneumonol Alergol Polska.* (1994) 62(Suppl. 2):39–41. 7894368

[17] Martins TB Bandhauer ME Bunker AM Roberts WL Hill HR. New childhood and adult reference intervals for total IgE. *J Allergy Clin Immunol.* (2014) 133:589–91. 10.1016/j.jaci.2013.08.037 24139495

[18] Sanjuan MA Sagar D Kolbeck R. Role of IgE in autoimmunity. *J Allergy Clin Immunol.* (2016) 137:1651–61. 10.1016/j.jaci.2016.04.007 27264000

[19] Maurer M Altrichter S Schmetzer O Scheffel J Church MK Metz M. Immunoglobulin E-mediated autoimmunity. *Front Immunol.* (2018) 9:689. 10.3389/fimmu.2018.00689 29686678 PMC5900004

[20] Sangle SR Tench CM D’Cruz DP. Autoimmune rheumatic disease and sleep: a review. *Curr Opin Pulm Med.* (2015) 21:553–6. 10.1097/MCP.0000000000000215 26402614

[21] Hsiao YH Chen YT Tseng CM Wu LA Lin WC Su VY Sleep disorders and increased risk of autoimmune diseases in individuals without sleep apnea. *Sleep.* (2015) 38:581–6. 10.5665/sleep.4574 25669189 PMC4355897

[22] Kang JH Lin HC. Obstructive sleep apnea and the risk of autoimmune diseases: a longitudinal population-based study. *Sleep Med.* (2012) 13:583–8. 10.1016/j.sleep.2012.03.002 22521311

[23] Foo YZ Nakagawa S Rhodes G Simmons LW. The effects of sex hormones on immune function: a meta-analysis. *Biol Rev Camb Philos Soc.* (2017) 92:551–71. 10.1111/brv.12243 26800512

[24] Quintero OL Amador-Patarroyo MJ Montoya-Ortiz G Rojas-Villarraga A Anaya JM. Autoimmune disease and gender: plausible mechanisms for the female predominance of autoimmunity. *J Autoimmun.* (2012) 38:J109–19. 10.1016/j.jaut.2011.10.003 22079680

[25] Salo PM Arbes SJ Jr. Jaramillo R Calatroni A Weir CH Sever ML Prevalence of allergic sensitization in the United States: results from the National Health and Nutrition Examination Survey (NHANES) 2005-2006. *J Allergy Clin Immunol.* (2014) 134:350–9. 10.1016/j.jaci.2013.12.1071 24522093 PMC4119838

[26] Leffler J Stumbles PA Strickland DH. Immunological processes driving IgE sensitisation and disease development in males and females. *Int J Mol Sci.* (2018) 19:1554. 10.3390/ijms19061554 29882879 PMC6032271

[27] Kalahasthi RB Rajmohan H Narendranan P Pradyumna A. Serum total immunoglobin-E and health hazards in workers involved in land fill and compost areas of hazardous waste management plants. *Ind J Occup Environ Med.* (2012) 16:9–13. 10.4103/0019-5278.99681 23112500 PMC3482711

[28] Whinnery J Jackson N Rattanaumpawan P Grandner MA. Short and long sleep duration associated with race/ethnicity, sociodemographics, and socioeconomic position. *Sleep.* (2014) 37:601–11. 10.5665/sleep.3508 24587584 PMC3920327

